# Supplementary material for: PIVOT: platform for interactive analysis and visualization of transcriptomics data
Source: BMC Bioinformatics. 2018 Jan 5;19:6. doi: 10.1186/s12859-017-1994-0 (PMC5756333; doi:10.1186/s12859-017-1994-0)
Supplement: Additional file 1: Table S1. — Comparison of tools integrated/implemented in PIVOT to other similar applications. (DOCX 80 kb) [file 12859_2017_1994_MOESM1_ESM.docx]

**Table S1**. Comparison of tools integrated/implemented in PIVOT to other similar applications.

| **Modules** | **PIVOT** | **RNASeqGUI** | **START** | **DEApp** | **ASAP** |
| --- | --- | --- | --- | --- | --- |
| **Supported Input** | Count/Expression matrix, Count folder, 10x Cell Ranger output folder, Sample metadata, PIVOT state | BAM file, Count/Expression matrix | Count/Expression matrix,  START RData file | Count matrix, Sample metadata | Count/Expression matrix, Sample metadata |
| **Normalization** | DESeq, Modified DESeq, TMM, Upper quartile, CPM/RPKM/TPM, RUV, Spike-in regression, Census | CPM, Upper quartile, TMM, Full quantile | CPM | CPM | Voom, DESeq, RPKM, scLVM, TMM, ComBat |
| **Feature/Sample Filter­ing** | QC plots; List based, Expression based and Quality based filters | QC plots; Expression based filter |  | Expression based filter | QC plots; Expression based and Quality based filters |
| **Basic Analysis Modules** | Data distribution plots, Dispersion analysis, Rank-frequency plot, Spike-in analysis, Feature heatmap, Gene expression visualization, etc. | Data distribution plots, Dispersion analysis,  Feature heatmap | Gene expression boxplots |  | Data distribution plots,  Feature heatmap |
| **Differential Expression** | DESeq2, edgeR, SCDE, Monocle, Mann-Whitney U test | DESeq2, edgeR,  NoiSeq, BaySeq | Voom | edgeR, DESeq2, Voom | Voom, edgeR, DESeq2, SCDE |
| **Clustering/**  **Classification** | Hierarchical, K-means, SC3, Community detection, Classification with caret, Cell state ordering with Monocle2/Diffusion Pseudotime |  |  |  | Hierarchical, K-means, SC3, PAM |
| **Dimension Reduction** | PCA, t-SNE, Metric/Non-Metric MDS, penalized LDA, Diffusion map | PCA | PCA | MDS | PCA, tSNE, MDS, ZIFA |
| **Correlation Analysis** | Pairwise scatter plots, Sample/feature correlation heatmap, Co-expression analysis and visualization | Pairwise scatter plots | Sample correlation |  | Sample correlation plots |
| **Gene Set Enrichment Analysis** | KEGG pathway analysis, Gene ontology analysis | KEGG pathway analysis, Gene ontology analysis |  |  | KEGG pathway analysis, Gene ontology analysis, Gene Atlas |
| **Network Analysis** | STRING protein association network, Regnetwork visualization, Mogrify based trans-differentiation factor prediction |  |  |  |  |
| **Other Utilities** | Data map, Gene ID/Name conversion,  BioMart gene annotation query, Venn diagram, Report generation, Program state saving | Venn diagram, Report generation, BAM exploration, Read count |  | DE result comparison, Venn diagram | Project management |
